# Number-action coupling across the lifespan: Sensorimotor foundations of numerical cognition

**DOI:** 10.1016/j.isci.2026.116420

**Published:** 2026-06-25

**Authors:** Swathi Prabhu, Michelle Giraud, Sara Noacco, Sabrina Brigadoi, Giovanna Mioni, Simone Cutini, Maria Silvia Saccani, Mariagrazia Ranzini, Elena Nava

**Affiliations:** 1 Department of Psychology, University of Milano-Bicocca, Milan, Italy; 2 Department of General Psychology (DPG), University of Padova, Padova, Italy; 3 Department of Developmental Psychology and Socialisation (DPSS), University of Padova, Padova, Italy

**Keywords:** Behavioral neuroscience, Cognitive neuroscience

## Abstract

A growing body of evidence suggests that aspects of numerical cognition interact with action systems, particularly hand motor control, although the strength and nature of this coupling vary across tasks, developmental stages, and cultural contexts. This narrative review synthesizes behavioral, developmental, comparative, and neurophysiological evidence to address four central questions: (1) how early the connection between number and action emerges and how it changes with development; (2) the extent to which education and culture shape number-action coupling; (3) how aging alters these processes and their neural substrates; and (5) what number-action interactions reveal about the broader organization of shared cognitive and sensorimotor mechanisms. Although the evidence remains heterogeneous, as causal demonstrations remain limited and effects are often task-specific, this review nevertheless suggests that number-action mappings are present from infancy, supported by overlapping parietal-frontal networks, and are progressively refined through motor experience. Across childhood, finger gnosis and fine motor skills scaffold numerical development, while cultural practices such as finger-counting shape enduring representational formats. In adulthood, numerical magnitude and action show a robust bidirectional influence, with converging neuroimaging evidence for shared fronto-parietal substrates. In older age, despite executive slowing and motor decline, number-action overlap appears preserved via compensatory recruitment, and embodied interventions show promise. Together, these findings support a layered account in which action systems contribute to scaffold and modulate numerical cognition across the lifespan.

## Graphical abstract

**Figure:**
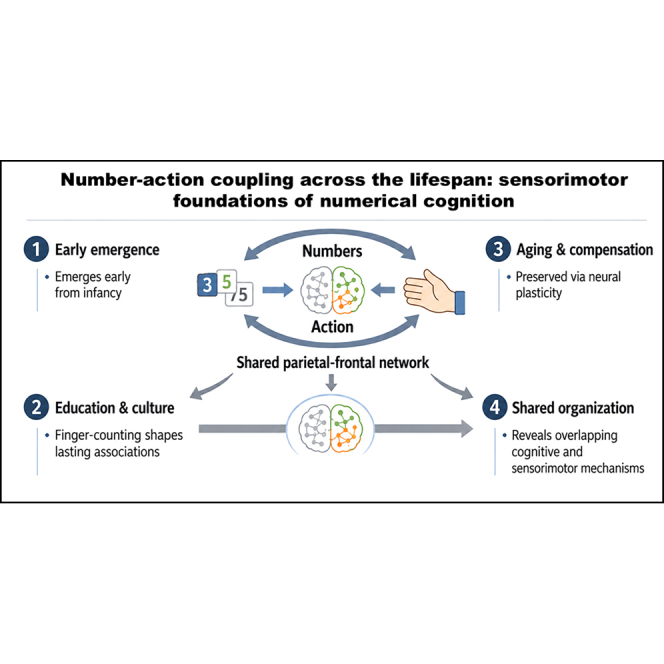

## Introduction

In everyday life, humans constantly interact with quantities that demand action: estimating distances, tracking sequences of movements, judging how many objects are present or coordinating actions over time. Such behaviors require rapid integration of numerical, spatial, and temporal information with motor planning and execution. Increasing evidence suggests that these domains rely on partially shared neural mechanisms, challenging traditional views of numerical cognition as a set of purely abstract and symbol-driven cognitive processes.^1^ This review examines number-action interactions across the lifespan, focusing on hand motor control as a privileged interface between cognition and action. It is important to note that our aim is not to argue that numerical cognition is uniformly or necessarily grounded in action systems; we rather synthesize evidence for multiple forms of number-action coupling that differ in strength, causal status and task dependence. Furthermore, we here use the term “action” to refer to multiple, partially dissociable levels of the motor system. Specifically, we distinguish between: (1) motor execution, referring to overt, goal-directed movements such as grasping or pointing; (2) motor planning and programming, including the selection and parameterization of actions prior to movement onset; (3) motor representations activated without overt movement, such as during action observation or affordance processing; and (4) culturally learned sensorimotor routines, such as finger-counting gestures.

Importantly, action is not used as a synonym of movement per se: some processes reviewed here involve covert, simulated, or observed actions rather than executed movements. We focus primarily on goal-directed hand actions, while also considering other effectors when they inform shared magnitude-action mechanisms.

We structure the review around four theoretical questions that cut across development and aging.

(1) How early does the number-action connection emerge, and how does it change with experience and maturation?
(2) To what extent do education and culture shape number-action coupling?
(3) How does aging alter these interactions and their neural bases?
(4) What do number-action interactions reveal about the broader organization of shared cognitive and sensorimotor systems?

This organization allows us to integrate developmental, cultural, and neurophysiological findings within a layered framework that preserves heterogeneity across mechanisms, tasks, and age groups.

## Early emergence and developmental trajectory of number-action coupling

### Evolutionary and theoretical foundations

The existence of a relationship between magnitudes and actions can be understood in light of evolutionary pressures that required organisms to act efficiently on quantitative information. Here, action primarily refers to goal-directed motor behaviors that require integration of magnitude information for planning and control, rather than simple reflexive movements.

Survival depended on judging how far away prey, predators, or mates were (space), anticipating when an event would occur or how long an action would take (time), and estimating how much food was available or how many competitors or enemies were present (quantity). Crucially, these magnitudes did not serve abstract judgments, but directly supported concrete actions, such as approaching, fleeing, or attacking.

Because magnitudes provide information that is immediately relevant for action, evolution may have favored the development of a shared neural coding system, rather than independent mechanisms for time, space, and quantity. This idea is formalized in a “Theory of Magnitude (ATOM),”^2^ which proposes that magnitude representation is fundamentally action-oriented. According to this theory, representations of time, space, and number evolved because they enhanced adaptive behavior, allowing organisms to efficiently integrate multiple dimensions of magnitude in the service of action.

Within this framework, different magnitude dimensions rely on a common cortical substrate, primarily located in the inferior parietal cortex. Supporting this view, converging evidence from neuroimaging, transcranial magnetic stimulation (TMS), and behavioral studies shows overlapping parietal activation during tasks involving time, space, size, and number.^3^^,^^4^ Functional magnetic resonance imaging (fMRI) studies further demonstrate that both non-symbolic numerical and spatial processing recruit overlapping regions of the right posterior parietal cortex in adults, whereas in children numerical processing additionally engages regions associated with finger use, such as the supramarginal gyrus and the precentral and postcentral gyri.^5^ Similarly, an fMRI study employing temporal, numerical, and spatial discrimination tasks revealed shared activation within a network including the intraparietal sulcus (IPS), insula, premotor cortex, and inferior frontal gyrus.^6^

Building on ATOM, the GradiATOM framework^7^ refines this account by proposing that magnitude processing is organized along cortical gradients across parietal and frontal regions. In its original formulation, GradiATOM showed that space and time recruit a shared fronto-parietal network, but with graded differences: spatial processing is associated with more dorsal parietal/frontal regions and anterior supplementary motor area (SMA) (pre-SMA), whereas temporal processing is associated with more ventral frontal/parietal regions and posterior SMA (SMA-proper). Subsequent work extending this framework to numerosity suggests that number processing overlaps with both space and time within this shared network, with space-number proximity observed mainly in parietal regions and SMA, and time-number proximity mainly in frontal regions.

Importantly, these theoretical frameworks predict in a different way the level at which number-action interactions arise. The ATOM theory proposes a common shared magnitude system, but it does not specify whether effects emerge at perceptual, decisional, or motor stages. Differently, GradiATOM suggests a graded organization spanning from low-level sensorimotor to higher level control processes across parieto-frontal hierarchies. In this framework, sensorimotor numerosity accounts more strongly predict low-level coupling, in which numerosity is directly represented in motor-relevant formats. A recent meta-analysis^8^ identified common loci activated across space-, time-, and number-related tasks, including the bilateral insula, SMA, right inferior frontal gyrus, and bilateral IPS. These findings reinforce the idea that magnitude processing relies on a domain-general system and is intrinsically linked to action planning and control.

One domain in which the interaction between action and perception is particularly evident is non-symbolic number processing, that is, the ability to perceive numerosity without relying on explicit counting.

Extending the ATOM framework,^9^ proposed a sensorimotor numerosity system, composed of channels tuned to specific numerical ranges that support the planning and execution of actions. In this view, numerosity perception and motor programming share a common coding system, allowing actions to be optimized based on quantitative information. Thus, while ATOM and GradiATOM describe a shared and gradient-based cortical architecture for magnitude processing, the sensorimotor numerosity account emphasizes a direct functional coupling between numerical information and action planning.

Comparative cognition provides further support for the evolutionary relevance of number-action links. Species ranging from primates and birds to fish and insects demonstrate abilities to estimate quantity, assess distances, and discriminate durations—skills essential for survival tasks such as predator avoidance, foraging, and social coordination.^10^ Neurophysiological studies in non-human primates show that neurons in the superior parietal lobule (SPL) encode the ordinal position of self-generated movements, effectively “counting” action repetitions.^11^ This mechanism may represent an evolutionary precursor of abstract numerical abilities, enabling the brain to process numerical information derived both from external stimuli and from internally generated actions using partially overlapping neural resources.

Taken together, evidence from theoretical models, neuroimaging, and comparative research suggests that number-action coupling is not a mere by-product of higher cognition, but might be a core feature of the magnitude system shaped by evolutionary pressures. Since perception and action are deeply intertwined in everyday behavior, a shared system capable of representing quantities in the environment and tracking self-produced actions (e.g., steps, taps, and gestures) would provide a clear adaptive advantage by integrating sensory input with motor output.

These findings are consistent with the possibility that some number-action links reflect early developing constraints within magnitude systems, while leaving open the extent to which such links are later shaped, strengthened, or replaced by experience, symbolic learning, and task demands.

Indeed, from a lifespan perspective, number-action interactions should not be attributed to a single mechanism. In neonates and young infants, these links likely arise at a basic sensorimotor level, reflecting broad correspondences between numerical magnitude and action-related parameters before executive functions are mature. Across childhood, these early couplings become progressively reorganized into more stable cognitive and representational mappings through motor experience, finger-based routines, symbolic learning, and cultural practices. Executive control therefore is not a prerequisite for the emergence of number-action interactions; rather, it constitutes a later-developing layer that modulates these mappings by supporting flexible selection, inhibition of competing responses, and adaptation to contextual demands. In aging, this executive layer may become especially important as a compensatory mechanism that helps preserve performance when lower-level sensorimotor or processing efficiency declines. Under this view, infancy and aging findings are complementary: early number-action links are foundational and pre-executive, whereas later executive processes refine, coordinate, or compensate for interactions that emerge earlier in development.

### Blueprints of the number-action relationship in infants

Babies are thought to demonstrate the ability to estimate and compare numerical quantities through a primitive system known as approximate number system (ANS), which enables humans and other species to discriminate quantities based on ratio. For example, newborns (1–4 days old) can match auditory and visual stimuli presented in a 3:1 ratio,^12^ and by 4–6 months of age, discrimination improves to a 1:3 ratio,^13^ gradually refining through development and schooling to near-adult acuity by adolescence (e.g., 10:11).^14^

Since the earliest stages of life, numerical information seems to be systematically linked to other magnitude dimensions relevant for action. Evidence in this regard comes from spatial-numerical associations (SNARC) observed in newborns: infants as young as 11 h to 5 days associate smaller numerosities with the left side of space and larger numerosities with the right, even when continuous variables such as cumulative area and perimeter are controlled.^15^^,^^16^These findings indicate that numerical magnitude is spontaneously mapped onto spatial coordinates, reflecting early emerging number-space associations. Comparable left-right numerical mappings have been documented in non-human species, including domestic chicks and Clark’s nutcrackers,^17^^,^^18^ suggesting a phylogenetically conserved, at least partly innate relationship between number and spatial representations.

Further support for an early experience-independent number-action link comes from cross-modal congruency effects: in the first months of life, infants preferentially match auditory numerosities with visual magnitudes such as line length or area.^19^^,^^20^ All these cross-modal correspondences suggest that numerical magnitude is embedded within a broader magnitude system that integrates perception and action well before formal learning or cultural input.

Critically, recent work demonstrates that the relationship between number and action in infancy is bidirectional. At 3–4 months of age, congruent pairings of numerosity and hand aperture (e.g., larger numerosities with larger apertures) elicit distinct electrophysiological responses compared to incongruent pairings.^21^ By 7–9 months, infants habituated to congruent mappings reliably detect violations when presented with incongruent number–action pairings, whereas the reverse is not observed, revealing an asymmetry that favors naturally aligned mappings.^22^ These effects are number-specific and cannot be reduced to general size processing, as they persist even when continuous magnitudes are controlled.^16^^,^^23^

**Figure 1:**
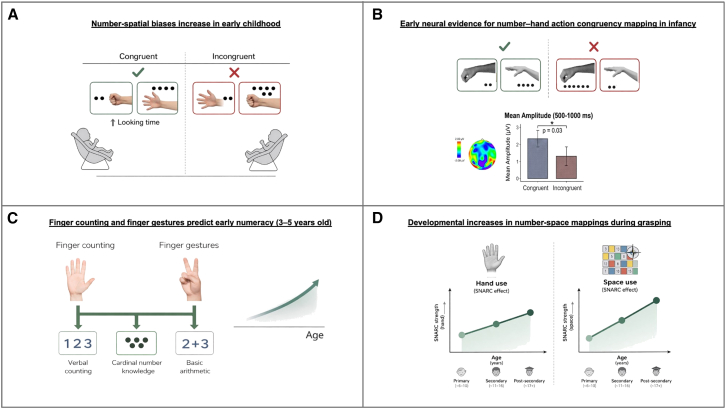
Developmental trajectory of number-action mappings from birth to childhood (A) Number-action mappings are already present at birth: human newborns preferentially associate numerical magnitude with action-related information, indicating that links between number and action emerge without extensive sensorimotor experience.^24^ (B) Electrophysiological evidence in infancy, showing that numerical magnitude modulates neural responses related to action processing, thereby confirming that number-action mappings are instantiated at the neural level early in development.^22^ (C) Early mappings become behaviorally expressed and functionally relevant in early childhood: finger counting and finger number gesturing in 3- to 5-year olds, both uniquely predict basic numerical skills, highlighting the role of embodied action in the development of formal number knowledge.^25^ (D) Numerical magnitude continues to bias action selection later in childhood and adulthood, as evidenced by an SNARC effect in a visually guided grasping task, with magnitude influencing hand and space selection during real-world actions.^26^

Recently, de Hevia and colleagues^24^ showed that newborns possess an abstract mapping between number and action from birth, rooted in sensorimotor representations rather than simple visual features. Across three experiments with 48 newborns, infants were shown changes in number alongside changes in hand aperture. Newborns looked longer at congruent pairings—where increases or decreases in number matched corresponding changes in hand opening—but only when the stimuli involved biological hands. This study reveals that action-number mappings appear to be present from the very beginning of postnatal life, suggesting a foundational role for sensorimotor processes in numerical cognition (Figure 1A).

The broader sensorimotor context in which these mappings emerge—namely, how infants progressively adapt their actions to physical magnitudes over the first year of life—has been comprehensively reviewed by Aychet et al.^27^ in a recent preprint. Their synthesis charts the development of manual action control from early pre-reaching movements to visually guided grasping, documenting how infants adjust grip aperture to object size, transition between uni- and bi-manual reaches, and refine finger and wrist coordination. They also review evidence that infants anticipate others’ grasping actions based on magnitude cues such as object size and distance. While this work primarily addresses continuous spatial magnitudes rather than discrete number, it provides an essential motor foundation on which number-action mappings can emerge. In the present review, we therefore build on this framework by focusing specifically on how and when numerical magnitude itself becomes integrated into the developing action-magnitude system, and how it interacts with the emerging motor repertoire.

At the neural level, early number-action integration is supported by shared parietal and motor networks. Electrophysiological evidence^22^ (see Figure 1B) points to overlapping substrates within the IPS and parieto-frontal circuits. By 8 months of age, infants show clear neural and behavioral preferences for congruent numerical-motor pairings,^22^^,^^28^ further confirming the early integration of numerical and physical dimensions. By 9 months, infants even appear capable of integrating numerical magnitude with rudimentary arithmetic operations. Notably, the strength of neural entrainment to numerical changes at 7 months predicts later behavioral performance in number discrimination tasks at 9 months, providing longitudinal evidence for early individual differences in numerical processing.^29^ Neural recordings further suggest that right parietal activity feeds forward to frontal error-detection systems, enabling early forms of magnitude-based evaluation and prediction.^30^ This pattern indicates that numerical processing recruits motor-related brain regions from the outset, particularly within right parietal cortex, even before language or formal mathematical instruction.

Behavioral evidence converges with these neural findings. Preverbal infants orient attention faster to the left when primed with small numbers and to the right when primed with large numbers,^31^ and they consistently prefer congruent number-space and number-action pairings across modalities.^12^ These results align closely with the ATOM framework,^2^ which posits a common magnitude-processing system linking number, space, and time—one that numerical cognition can exploit from the earliest stages of development.

In summary, evidence from behavioral, electrophysiological, and neuroimaging studies indicates that the number-action relationship is already present in infancy, emerges prior to formal education or cultural shaping, and is expressed bidirectionally. These paradigms do not involve overt motor execution by the infant, but rather perceptual sensitivity to action-related parameters (e.g., hand aperture); thus, they should be interpreted cautiously because they do not establish that action is causally necessary for numerical representation. Nevertheless, they represent the window into understanding how these early mappings are then refined through motor development and supported by shared parietal and motor circuits, providing a foundational scaffold upon which later symbolic, culturally mediated number-action interactions are built.

### Number-action relationship in childhood: The role of experiences

Children build numerical understanding through active, body-based exploration of the world. From infancy onward, they learn to count, sort, and compare quantities by physically interacting with objects—touching, grasping, and manipulating sets of items. These behaviors primarily involve goal-directed hand actions, encompassing both motor execution (e.g., grasping, manipulating objects) and motor planning (e.g., sequentially organizing counting actions). A large body of evidence shows that these early sensorimotor experiences are systematically related to later numerical competence. Barrocas et al.,^32^ for example, reviewed decades of work demonstrating that both finger gnosis (the ability to recognize and differentiate one’s own fingers through tactile information) and fine motor skills (FMS) predict children’s counting abilities, number magnitude understanding, and arithmetic performance. In everyday contexts, this link is most evident when young children literally “count on their fingers.” Beyond arithmetic, early numerical competence grounded in action can also support social reasoning, as shown by findings that preschoolers’ numerical understanding mediates age-related improvements in fair sharing.^33^

Formal schooling further strengthens links between number, space, and action. Within the first two years of education, children begin to show adult-like SNARC, mapping smaller numbers to the left and larger numbers to the right.^34^ Numerosity also biases hand use in reaching tasks, as revealed by Mills et al.^26^ (Figure 1D). Across 84 participants from primary school to adulthood, number magnitude systematically biased motor behavior during goal-directed reaching actions, affecting action selection (which hand to use) and aspects of motor execution. The findings provide strong evidence that number processing is tightly linked to sensorimotor systems, supporting embodied and action-based accounts of numerical cognition.^26^

Longitudinal evidence indicates that these domains develop in parallel: Liu and Zhang^35^ found that in 3-4-year olds, higher spatial ability predicted later gains in counting range and backward counting. Thus, from the preschool years onward, numerical knowledge and action systems—particularly spatial processing and motor coordination—seem to develop concurrently rather than independently, linking number representations with both motor planning and execution processes.

A key developmental transition during childhood concerns the internalization of counting strategies. Children initially count by pointing to physical objects, then by using their fingers, and eventually by relying on internal, abstract representations of numerosity. During this shift, children form mental “hand structures” derived from their finger-counting routines. Domahs and colleagues^36^ showed that German children frequently make “split-five” errors (e.g., representing 8 as 5 + 3), reflecting an implicit base-5 structure rooted in hand use. Strikingly, similar effects persist into adulthood,^37^ and adults show slower or less accurate numerical performance when finger use is experimentally prevented.^38^ These findings indicate that finger-based representations do not merely scaffold early calculation but can leave durable traces in internal number representations, consistent with embodied and neural redeployment accounts of cognitive development.^36^^,^^39^

Importantly, Roesch and colleagues^25^ added important findings to previous notions, by showing that spontaneous finger use is highly context-sensitive, i.e., children rarely use finger counting spontaneously but frequently use finger number gestures in socially familiar contexts (e.g., showing their age). Furthermore, the authors found better performance in prompted finger counting than finger number gesturing, suggesting that finger counting may scaffold the later use of finger number gestures, supporting a sequential or hierarchical relationship between the two strategies. By linking distinct sensorimotor strategies to multiple numerical competencies, the findings strengthen theories of embodied cognition, suggesting that numerical understanding is grounded in bodily actions from early childhood (Figure 1C).

As children gain finer motor control, their numerical skills improve in parallel. Recent reviews confirm robust associations between motor abilities and early mathematics. Flores and colleagues,^40^ for example, showed that visuomotor integration (hand-eye coordination) was the strongest predictor of preschool math performance, with gross motor skills contributing indirectly by supporting visuomotor coordination. In this sense, motor development does not directly drive math skills but provides a functional basis for numerical learning.

Finger-specific abilities appear to play a particularly important role. Numerous studies report that children with better finger awareness and dexterity show stronger early numerical skills. In a large sample of 153 children aged 3–5 years, Fischer and colleagues^41^ found that FMS predicted finger-based counting and calculation, while finger gnosis predicted finger monitoring; neither predicted number-line estimation, suggesting task-specific rather than global effects. Finger gnosis is also linked to both behavioral accuracy and neural efficiency: Zhang and colleagues^42^ showed that 5-6-year olds with better finger gnosis solved addition problems more accurately, while neuroimaging evidence indicates that higher finger gnosis is associated with reduced activation in number-processing regions, suggesting more efficient neural processing.^43^ These findings highlight the role of fine-grained motor representations of the hand, even in the absence of overt movement.

Importantly, the contribution of finger gnosis to arithmetic is reliable but modest. Large-scale studies suggest that finger gnosis explains only a small proportion of variance in later arithmetic performance once general intelligence and early numerical skills are controlled. For instance, Wasner and colleagues^44^ reported that finger gnosis uniquely accounted for only 1%–2% of variance in first graders’ arithmetic, and Poltz and colleagues^45^ found similarly weak effects after controlling for age and IQ. Taken together, these findings indicate that finger gnosis and FMS support numerical development most strongly for tasks that explicitly rely on finger-based strategies,^41^ highlighting the need for longitudinal and comparative designs to clarify causal mechanisms.

### Cultural shaping of embodied number representations

Children across cultures spontaneously use their fingers when learning number concepts, likely in response to how adults highlight quantities. However, the specific ways in which fingers are used vary markedly across cultures. Embodiment accounts describe fingers as an early cognitive “tool” for numbers,^46^ and cross-cultural research documents substantial variation in finger-counting conventions.^47^^,^^48^ Many Western children use a two-handed system (1–5 on one hand, 6–10 on the other), whereas Chinese children typically use a one-handed system with symbolic gestures for numbers above five.^42^ These differences have measurable neural consequences: simply viewing Arabic digits activates motor cortex, and the hemisphere involved depends on an individual’s counting habits.^49^

Crucially, culturally learned finger-counting routines influence arithmetic performance. Western children are slower and less accurate on problems that cross the five-finger boundary (e.g., 4 + 3), reflecting the cognitive cost of switching hands in their internal counting scheme. Chinese children, who use a single-hand system, do not show this sub-base-5 cost.^42^ These findings demonstrate that while embodiment provides the mechanism, specific actions, like overt structured motor routines (e.g., counting) are culturally established leading to predictable differences in how numerical and motor circuits interact.

This enculturated embodiment challenges strong claims of universal spatial-numerical mappings. The same embodied learning process can yield different internal representations depending on cultural practice. By late childhood, these habits become internalized: even passive number perception automatically activates sensorimotor regions corresponding to an individual’s counting history.^46^^,^^50^ Thus, number-action links are not fixed but emergent and plastic.

### The role of training and intervention

Intervention studies provide direct evidence—yet limited—that motor experience can influence some numerical skills, especially training programs, especially those that emphasize finger use or FMSs. These interventions primarily engage goal-directed hand actions, involving both motor execution (e.g., finger movements during counting) and motor planning (e.g., sequencing and structuring numerical operations). Artemenko and colleagues^50^ showed that first graders who engaged in daily finger-counting games for 1 year developed stronger sub-base-5 effects and increased sensorimotor cortex activation during arithmetic. Similarly, Asakawa et al.^51^ found that a brief three-week fine-motor training program led to significant gains in arithmetic compared to a reading control group.

Long-term training provides converging evidence. Intensive abacus-based mental calculation (AMC) training, which combines finger movements with mental imagery, leads to reorganization of functional brain networks, with stronger sensorimotor and visual connectivity and reduced reliance on fronto-parietal control regions.^52^^,^^53^ These findings suggest that sustained motor engagement can shift arithmetic processing toward more autonomous, embodied neural solutions.

Most interventions target early childhood, and the motor-math link appears strongest during this period. By adolescence, arithmetic becomes increasingly abstract, and overt motor involvement diminishes. Nonetheless, residual motor influences persist into adulthood, including interference from finger movements and spatial biases.^38^ The adolescent transition remains underexplored but is likely to reflect increasing internalization of number-action links under formal education.

Computational embodied models also provide mechanistic support for these findings. Simulated agents acquire numerical representations through repeated action-perception loops, learning number concepts by predicting action outcomes, and integrating sensorimotor feedback.^54^^,^^55^ In this context, “action” refers to goal-directed interactions with the environment that couple motor output with sensory consequences. These models reinforce the idea that numerical knowledge can emerge from action rather than symbolic instruction alone.

In sum, number-action relationships in childhood are inherently embodied yet highly sensitive to experience. Motor development—particularly finger use—supports numerical learning, while education and culture shape which actions become linked to which numbers. The relationship is bidirectional: numerical tasks recruit motor circuits, and motor training sharpens numerical processing. Children learn numbers through both overt actions and their progressive internalization, but their environment determines how those actions are structured and internalized.^32^

Together, these studies provide an example of a continuous developmental pathway in which number-action mappings emerge at birth, are supported by early neural mechanisms, are strengthened through embodied strategies such as finger use, and remain functionally integrated with action systems across development.

### Atypical populations as a window into the role of action in number: Action mapping

Before entering the second part of the review, an important issue that can provide a strong theoretical clue to number-action mappings, is represented by atypical populations. Indeed, from an embodied cognition perspective, if numerical representations are grounded, at least in part, in action-based experiences, then motor impairments should have measurable consequences for numerical development, while numerical impairments may also reveal alterations in motor-related processes. Evidence from atypical populations is therefore particularly valuable because it helps clarify not only whether number and action systems interact, but also whether such interactions are necessary, functional, or only epiphenomenal, one route among several possible developmental pathways.

Consistent with this view, studies of children with motor impairments suggest that altered motor development can affect early mathematical learning. In typically developing children, FMSs, finger gnosis, and visuomotor integration are associated with numerical competencies such as counting, magnitude comparison, and arithmetic.^41^^,^^56^^,^^57^^,^^58^ In atypical development; however, children with motor impairments, such as developmental coordination disorder (DCD) or cerebral palsy, can show delays or difficulties in counting, number sequencing, and basic arithmetic.^59^^,^^60^ These difficulties have been linked to impairments in visuospatial processing, procedural learning, and action sequencing, all of which are thought to support the acquisition of numerical concepts. Children with DCD, for example, show reduced performance in tasks requiring ordered action execution and temporal coordination, which may directly affect the development of ordinal numerical representations and counting routines.^60^ Similarly, in cerebral palsy, limitations in hand function and reduced opportunities for object manipulation may constrain early numerical learning experiences, particularly those relying on embodied interactions such as finger counting or set manipulation.^61^^,^^62^

Studies of finger representation also offer a unique opportunity to understand the role of the sensorimotor system in numerical cognition. For instance, classic developmental studies have shown that reduced finger awareness is associated with weaker arithmetic performance and less efficient use of finger-based strategies.^58^^,^^63^^,^^64^ Another interesting case for the study of number-finger mapping, though not extensively investigated yet, concerns signed languages, in which numerical information is not only conveyed symbolically but is often embedded within the visuo-manual structure of the language itself.^65^

At the same time, atypical populations also reveal important limits to strong necessity claims. Not all individuals with motor impairments show severe numerical deficits, and some develop compensatory strategies that rely more heavily on verbal or symbolic processing.^66^ This pattern suggests that motor experience may facilitate and structure numerical development but is not strictly necessary in an absolute sense. Rather than implying that action is invariably required for all forms of numerical cognition, these findings support the view that action provides an important developmental scaffold that can, under some conditions, be complemented or partially replaced by alternative cognitive routes.

Conversely, research on individuals with numerical impairments provides insight into the motor side of the relationship. Developmental dyscalculia has been associated not only with deficits in numerical magnitude processing but also with impairments in spatial, attentional, and, in some cases, motor domains.^67^^,^^68^ Several studies report that children with dyscalculia show reduced finger gnosis, poorer fine motor coordination, and atypical use of finger-based strategies compared with typically developing peers.^58^^,^^69^^,^^70^ Deficits in motor sequencing and timing have also been observed, suggesting a partial overlap between numerical and motor control systems.^71^

Similarly, neuropsychology often shows co-occurrence of numerical and motor-related impairment following parietal brain lesion. For instance, neuropsychological findings in individuals with Gerstmann syndrome show co-occurring impairments in finger gnosis, calculation, and spatial orientation,^72^^,^^73^ and this has been traditionally interpreted as evidence of partially shared parietal substrates for motor and numerical processing. This hypothesis has been debated, and recent evidence indicates that this syndrome might arise from a disconnection of tracks connecting different parietal regions and not from the disruption of a common neural area.^72^^,^^74^ Nevertheless, dyscalculia and acalculia represent unique opportunities to explore the effects of disruption on the integration of the numerical and the action systems.

That said, not all individuals with dyscalculia present motor impairments, and some exhibit intact motor coordination despite severe numerical deficits.^75^ Such findings are theoretically important because they indicate that number and action systems are not mandatory coupled. Instead, their relationship appears to depend on developmental timing, task demands, and the specific components of motor and numerical processing being assessed. This pattern argues against both a fully modular account and a strong claim that action is always necessary for numerical cognition. Rather, it supports a layered framework in which action-related processes are developmentally important and often constitutive, but not uniformly indispensable for every form of number processing. In sum, atypical populations provide a particularly informative way to evaluate competing theoretical accounts. Strong embodied views would predict that disrupting motor experience, especially early in development, should lead to systematic impairments in numerical cognition. Hybrid or layered accounts instead predict partial dependence: motor systems scaffold the emergence of numerical representations, but these representations may later become increasingly supported by symbolic, verbal, or domain-general mechanisms. Modular accounts, in contrast, predict greater independence between domains. Patterns of co-occurrence, compensation, and selective dissociation in atypical populations can therefore help constrain these theoretical alternatives and clarify which aspects of number-action coupling are foundational, which are facilitative, and which may reflect broader shared developmental constraints.

## Number-hand interactions in adulthood

Adults frequently use hand gestures to convey numerical information even when quantity could be communicated verbally alone—for example, when ordering two glasses of wine or enumerating points during an argument. Such gestures are not merely communicative embellishments but reflect a persistent coupling between numerical meaning and action systems. This coupling is observable not only at the behavioral level but also within the neural networks supporting number processing and motor control. Although few studies have explicitly manipulated both domains within a single experimental design, converging evidence across paradigms supports the conclusion that number-action interactions in adulthood are bidirectional, with numerical magnitude influencing motor behavior and motor activity, in turn, shaping numerical processing.

### Behavioral evidence for bidirectional number-action interactions

**Figure 2:**
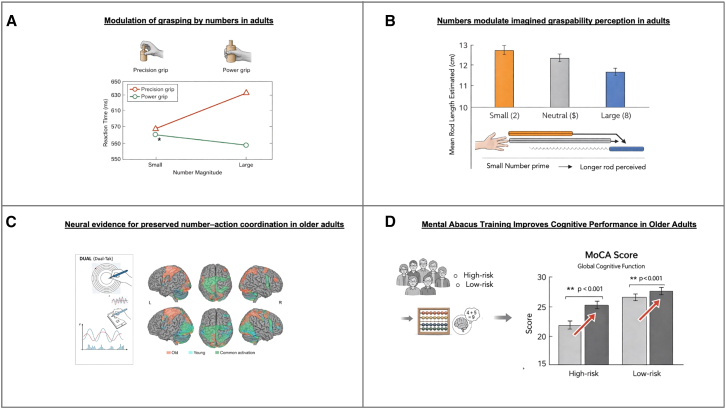
Interactions between numerical magnitude, action, and cognition in adulthood and aging (A) Numerical magnitude directly influences motor execution: viewing small versus large numbers primes different grasp apertures during object grasping, demonstrating a tight coupling between number processing and action parameters.^76^ (B) Numerical magnitude modulates action judgments even in the absence of overt movement, indicating that number-action links operate at a representational level.^79^ (C) Numerical processing and motor action do not compete for shared brain resources in aging, suggesting age-related reorganization of shared cognitive-motor resources.^80^ (D) Intensive mental abacus training—an embodied numerical practice—can improve cognitive function in older adults, potentially by reinforcing number-action representations (Huang et al.^81^).

Consistently with a low-level sensorimotor coupling, characterized by a direct mappings between numerical magnitude and action parameters, strong evidence comes from studies examining how numerical magnitude primes motor planning and execution of goal-directed hand actions. Using precision versus power grips as response modalities, several studies have shown that small numbers facilitate faster initiation of precision grips, whereas large numbers facilitate power grips^76^ (Figure 2A). Importantly, this effect persists even when numerical magnitude is irrelevant to the task, suggesting automatic activation of motor representations by numerical information.^77^ Similarly, Andres et al.^78^ demonstrated that small numbers speed grip closure, while large numbers facilitate grip opening, supporting the idea of shared representations between numerical and physical magnitudes.

Crucially, numerical interference is strongest during motor planning rather than execution, consistent with Glover’s (2004)^82^ planning-control model. In line with this account, Andres et al.^83^ showed that numerical magnitude modulated grip aperture primarily during the early phases of grasping, with the effect diminishing during movement execution. Numerical magnitude also biases judgments about potential actions: small versus large numbers influence graspability judgments even when no overt movement is performed, suggesting effects on covert motor representation (e.g., affordance processing) but not overt motor execution itself^79^ (Figure 2B). Crucially, number-action interactions can also emerge in the absence of an explicit motor response: numerical magnitude can modulate spontaneous grip force, with smaller numbers increasing left-hand force and larger numbers increasing right-hand force.^84^ Similarly, previous studies have shown that object affordance can emerge also in absence of implicit processing and interact with numerical magnitude.^85^

Extending beyond grasping, Rugani et al.^86^ used a finger-kick paradigm and showed that numerical magnitude affected both action selection and kinematics: small numbers induced leftward deviations, while large numbers improved trajectory efficiency. Notably, these effects were absent for non-symbolic numerosities, reinforcing the view that symbolic numbers engage spatial-motor systems more strongly than approximate quantity representations.

Taken together, these findings indicate that numerical magnitude can systematically shape multiple levels of action including motor planning, selection, and execution, even when numbers are task-irrelevant.

Evidence for the reverse direction—action to number influences—is less abundant but conceptually critical. Sequential finger movements (i.e., overt, goal-directed motor execution) selectively interfere with addition and subtraction, but not multiplication, suggesting that motor activity disrupts arithmetic operations relying on counting-based strategies rather than memory retrieval.^38^^,^^87^ This selective interference implies a functional link between finger-related motor processes and specific numerical operations that persists into adulthood. Action affordances further modulate number processing: viewing graspable objects enhances numerical magnitude effects (faster responses for small numbers), an effect absent for non-graspable objects,^85^ indicating that covertly activated motor representations can sharpen numerical sensitivity.

Recent work further suggests that different motor actions interact with distinct dimensions of number meaning. Repetitive pointing movements selectively modulate number comparison, slowing responses and particularly affecting small-number processing.^88^ This pattern supports the proposal that grasping is preferentially linked to cardinal magnitude, whereas pointing—rooted in culturally learned counting routines—engages ordinal representations.^89^ Beyond hand actions, continuous, goal-directed movements involving different effectors, like whole-body motion also influences numerical cognition: leftward or downward movement biases number generation toward smaller values, whereas rightward or upward movement biases responses toward larger numbers.^90^ These findings demonstrate that even vestibular input can interact with numerical representations via shared spatial-motor codes.

Overall, although evidence for action to number remains less abundant than number to action, the current literature provides support to a bidirectional interaction between numerical magnitude and motor systems in adulthood, spanning overt and covert, discrete and continuous, and planning-versus execution-level processes, consistent with an embodied view of numerical cognition.

### Neural substrates of number-action interactions

Neuroimaging evidence corroborates behavioral findings by revealing overlapping neural substrates for numerical processing and different level of the motor system, including motor planning, execution, and representation. Numerosity processing in adults relies on both shared and specialized mechanisms within the IPS, a region central to the integration of numerical, spatial, and temporal information. Parietal areas interact with frontal motor regions to support flexible, context-dependent behavior, consistent with a domain-general magnitude system supporting both decision-making and action.^91^ The anatomical proximity between numerosity-selective regions and motor-related areas within parietal cortex provides a structural basis for number-action interactions.

Anobile et al.^92^ formalized this idea by proposing an active sensorimotor numerosity system, in which numerosity estimation is intrinsically linked to voluntary action. In this framework, medio-temporal areas encode numerical information, while superior and medial portions of the inferior parietal cortex process non-symbolic quantities and numerical aspects of observed actions. Meta-analytic evidence further supports this view. Ranzini et al.^88^ identified converging activation for number comparison and hand actions (e.g., grasping, reaching) within bilateral IPS, anterior IPS (aIPS), superior parietal lobule (SPL), SMA, and left precentral gyrus. Notably, overlap in aIPS extended across grasping, reaching, and pointing, suggesting shared coding for both discrete and continuous magnitudes. These results have been confirmed and extended by a recent fNIRS study (fNIRS: functional, near infrared spectroscopy) where the participants executed motor and numerical tasks in a within participants design.^93^

Causal evidence reinforces these associations. Disrupting the left angular gyrus with rTMS impairs both finger recognition and numerical processing in the same individuals, indicating shared neural resources for finger related covert motor representation and number cognition. Moreover, a distinct parietal cluster in the postcentral sulcus has been identified for estimating the number of observed manipulative actions, such as repeatedly moving or releasing an object.^94^ Adaptation studies further show that altering motor processing can distort numerical perception, implying a common representational substrate linking action and numerosity.^95^

### Cultural modulation of number-action coupling

Although number-action coupling in adulthood relies on stable neural networks, its specific expression is shaped by culturally structured sensorimotor routines. Finger counting, while widespread, varies in starting hand, finger sequence, and symbolic structure across cultures^96^ and is influenced by individual factors such as handedness.^97^ These routines represent learned action sequences that bridge motor execution and abstract numerical representations. Moreover, these culturally learned mappings reflect context-dependent associations between number and action, consistently with higher level cognitive-decisional mechanisms. These variations have measurable cognitive consequences. Morrissey et al.^47^ showed that Canadian adults using two-handed counting systems were slower when comparing numbers requiring both hands, whereas Chinese adults using one-handed systems showed no such cost. Similarly, Domahs et al.^39^ reported stronger sub-base-five effects in German participants than in Chinese participants, reflecting enduring influences of early finger-counting habits.

More radically, Yupno adults from Papua New Guinea—who rely on a body-based counting system—show difficulties in number-line tasks unless exposed to Western schooling.^98^ These findings demonstrate that spatial-numerical mappings are not universal end-states but culturally shaped outcomes of embodied learning. Thus, while adulthood is often assumed to reflect fully abstract number representations, motor, and cultural traces of early embodied experiences remain detectable and functionally relevant.

In sum, in adulthood, numerical cognition and motor systems remain tightly and bidirectionally linked. Numerical magnitude influences different aspects of action such as motor planning, selection, and execution, while actions like motor activity, affordances, and bodily motion shape numerical processing. These interactions are supported by shared fronto-parietal networks and are consistent with domain-general magnitude frameworks such as ATOM. Crucially, cultural experience continues to modulate how number-action couplings are expressed, demonstrating that embodied numerical cognition is both stable and plastic across the lifespan.

## Growing older: Changes in number-action interactions

### Cognitive aging and numerical representations: Preservation with changing control

Aging is accompanied by functional and physical changes that affect both cognition and action, offering an important test case for the stability of number-action interactions across the lifespan. Here, action primarily refers to goal-directed behaviors involving both motor planning and execution, which may be either overt (e.g., manual responses) or covertly simulated during cognitive tasks. Among the earliest cognitive abilities to decline are executive functions, including planning, strategic reasoning, and inhibitory control.^99^^,^^100^^,^^101^ These abilities depend heavily on frontal brain regions, which show earlier and more pronounced vulnerability to aging than posterior regions, as demonstrated by postmortem and neuroimaging studies.^102^ Although quantity processing and numerical magnitude representation are primarily associated with parietal cortex, executive functions such as working memory and inhibitory control are closely intertwined with numerical task performance.^103^ Evidence regarding the maintenance of the ANS in aging is mixed: while some studies report declines in non-symbolic numerical discrimination,^14^ others suggest a relative resilience of ANS acuity across the lifespan, with observed performance differences being attributable to declines in more general cognitive processes that support number processing.^104^^,^^105^ Tasks assessing ANS acuity often place substantial demands on these domain-general processes, particularly when non-numerical visual features must be suppressed or when numerical information must be maintained across brief delays. Consequently, apparent declines in ANS performance in older adults may arise from difficulties in the control and use of numerical information during motor planning and preparation rather than from impairments in the underlying numerical representation itself.

For symbolic numerical skills, a more consistent profile emerges. Older adults often show preserved or even improved accuracy, accompanied by slower response times.^105^^,^^106^^,^^107^^,^^108^ Formal symbolic skills—such as reading and writing Arabic numerals, mental calculation, and knowledge of arithmetic rules—are strongly influenced by individual variables, particularly years of education and accumulated experience.^109^ Strategy use also changes with age: in subtraction tasks, older adults are slower in encoding numbers and producing results but faster in executing procedures such as borrowing.^110^ More broadly, they show greater variability in strategy selection and require more time for metacognitive evaluation, although lifelong arithmetic experience often mitigates declines, allowing for reasonably efficient performance.^111^ Interestingly, older adults are often less affected by the numerical distance effect, suggesting more refined magnitude representations, possibly consolidated through lifelong practice.^107^^,^^108^ Thus, aging appears to preserve core numerical knowledge while altering the balance between efficiency, strategy, and executive control.

### Motor aging, neural compensation, and implications for number-action coupling

Age-related changes are also evident in the motor domain, with clear relevance for number-action interactions. Older adults typically show slower and longer hand movements, likely reflecting compensatory strategies for visual or proprioceptive decline.^112^ Fine motor precision is particularly affected in the non-dominant hand, and grasping movements—especially in dynamic contexts—are characterized by longer initiation times and wider grip apertures, reflecting changes in both motor planning and online control of movement execution.^113^^,^^114^ In motor planning tasks, older adults, like children, tend to plan grasps less in advance and often adopt uncomfortable final postures.^115^ Difficulties in modulating precision grip force persist even after training.^116^ At the neural level, better performance in fine motor tasks correlates with greater overall brain volume and reduced white matter lesion load.^117^ Despite widespread age-related gray and white matter loss, substantial inter-individual variability exists, and motor decline is far from uniform across older adults.

Neuroimaging studies of numerical processing further point to compensatory reorganization rather than simple decline. Huang et al.^118^ showed that older adults recruit parietal cortex bilaterally during numerical magnitude and physical size discrimination tasks, whereas younger adults show more lateralized activation. This bilateral recruitment appears compensatory, as it is associated with better task performance. Similarly, in numerical inductive reasoning tasks, older adults activate a broader set of cortical regions and show reduced deactivation of task-irrelevant areas, resulting in comparable accuracy but slower responses relative to younger adults.^119^ These findings align with broader models of cognitive aging emphasizing neural dedifferentiation and compensatory recruitment, and they suggest that number-action coupling may be maintained through the engagement of additional neural resources.

### Preserved integration, embodied training, and open questions in aging

Although no studies have directly examined the relationship between FMSs and numerical abilities in older adults, converging evidence suggests that shared number-action systems remain functional in later life. This is illustrated by dual-task studies combining numerical and motor demands. Van Impe et al.^80^ (Figure 2C) showed that both younger and older adults activate overlapping fronto-parietal regions—including SMA/pre-SMA, anterior insula, and left inferior precentral gyrus—during numerical tasks. While older adults recruited more extensive networks during motor tasks, performance in dual-task conditions did not differ between age groups, indicating efficient integration between numerical and motor processes rather than mutual interference. Similarly, Zhang et al.^42^ found that walking slightly slowed numerical processing in older adults without reducing accuracy, suggesting that engaging motor networks does not necessarily impair numerical computation. Together, these findings support the idea that number-action integration may play a stabilizing or compensatory role in aging rather than constituting a source of dual-task cost.

From an applied perspective, interventions that re-engage embodied numerical processing appear promising. While finger counting is primarily associated with childhood learning, tools such as the abacus require coordinated fine motor activity and numerical manipulation and have shown cognitive benefits in older adults. Huang et al.^81^ demonstrated that abacus-based training improved executive functions (inhibition, planning), processing speed, sustained attention, and numerical working memory. These results suggest that reactivating sensorimotor numerical representations later in life may strengthen both numerical and domain-general cognitive functions. Future work should directly test whether motor-based numerical training yields advantages over purely cognitive interventions and clarify whether action facilitates or interferes with numerical processing in aging. Addressing these questions has important theoretical implications for shared cognitive-sensorimotor systems and practical relevance for designing motor-cognitive interventions to support numerical functioning in the elderly population.

Together, the panels illustrate that numerical cognition can interact with sensorimotor systems across adulthood and aging, although the available evidence in aging is currently limited with respect to the adult and also developmental literature. Nonetheless, they suggest that numerical cognition and sensorimotor systems dynamically interact across the lifespan, thus revealing a very plastic nature of such interactions.

## Conclusions: What number-action interactions reveal about shared cognitive and sensorimotor systems

Number-action interactions extend well beyond merely documenting an association between numerical cognition and movement; they expose how cognition is organized in a brain built for perception-action control. Across infancy, childhood, adulthood, and aging, the literature reviewed here converges on a central claim: numerical cognition is embedded within a broader architecture that integrates magnitude processing with sensorimotor control, and this architecture is both early emerging and experience-shaped. Number is not merely represented in a sensorimotor brain; it is harnessed by circuits that evolved to select actions, predict outcomes, and coordinate behavior across multiple levels of the motor system, including planning, execution, and internally represented actions.

We highlight four main concepts that emerged from the present review place its finding within a broader conceptual framework.

(1) Number-action coupling may reflect action-oriented computational constraints.

ATOM and related accounts propose that magnitude representations (space, time, number) are action-oriented adaptations supported by partially shared cortical resources.^2^^,^^120^ The lifespan evidence reviewed here supports this view in a functionally specific way: numerical magnitude behaves like an input to motor system processes involved in action selection and control. In adults, symbolic numbers reliably bias grasp-related responses—small numbers speeding precision grips and grip closure, larger numbers facilitating power grips or grip opening.^76^^,^^77^^,^^78^ Critically, effects often peak during movement planning rather than execution, consistent with the idea that magnitude information constrains action parameterization at the stage where motor programs are selected and scaled.^82^^,^^83^ These planning-sensitive effects are difficult to explain if number were a purely abstract representation that only later “feeds into” motor output; instead, they indicate that numerical meaning can be encoded in formats that can be readily used for control.

The bidirectionality of number-action effects further strengthen the “action-first” interpretation. Sequential finger movements selectively disrupt addition and subtraction, but not multiplication—suggesting that motor activity interacts with strategy-dependent numerical processes that rely on counting-based procedures rather than rote retrieval.^38^^,^^87^ Even action affordances modulate number processing: viewing graspable objects enhances magnitude-related response patterns that are absent for non-graspable objects.^85^ Together, these findings support the claim that numerical cognition is integrated into perception-action loops rather than being separable from them.

(2) Shared systems are layered, not unitary.

A second key lesson from number-action interactions is that shared cognitive-sensorimotor mechanisms are neither fully domain-general nor fully modular; they appear layered, with partially overlapping hubs and task-sensitive routes. Neuroimaging studies show common recruitment of parietal and premotor regions across time, space, and number tasks^3^^,^^4^^,^^6^ and meta-analytic evidence support convergence in loci such as bilateral IPS, SMA, insula, and right inferior frontal regions.^77^ Developmental fMRI adds an important nuance: children recruit number-related networks that more strongly implicate finger-associated regions such as supramarginal gyrus and pre/postcentral gyri compared to adults,^5^ implying that the “shared” architecture is developmentally reweighted as strategies become internalized.

At the same time, behavioral dissociations show selectivity within this overlap. Different motor acts may connect to different dimensions of number meaning: pointing—rooted in ordinal sequencing routines—selectively modulates number comparison and disproportionately affects small-number processing,^88^ aligning with proposals that grasping preferentially engages cardinal magnitude, whereas pointing engages ordinality.^89^ Whole-body motion biases number generation in directionally consistent ways,^90^ implying that vestibular-spatial codes can modulate numerical production. These patterns argue against a single “common code” account and instead suggest a family of mappings supported by shared fronto-parietal infrastructure but recruited differently depending on representational format (symbolic vs. non-symbolic), task demands, and embodied routines.

Framework refinements support this layered view. GradiATOM proposes gradient transitions across parietal-frontal cortex, with numerical information bridging spatial and temporal processing streams.^7^^,^^8^ The sensorimotor numerosity proposal further specifies that numerosity perception can be directly tied to action planning via tuned channels supporting motor optimization.^92^^,^^95^ Meta-analytic overlap between number comparison and hand actions (grasping/reaching) in bilateral IPS, aIPS, SPL, SMA, and left precentral gyrus provides a plausible substrate for such mappings.^88^ Causal evidence complements these correlational findings: disrupting left angular gyrus impairs both finger gnosis and number processing, indicating shared neural resources linking finger representations and numerical operations.^121^

(3) Development shows abstraction is built from sensorimotor scaffolds.

A third insight comes from infancy and childhood: number-action coupling emerges early but is then elaborated through sensorimotor development and learning. Infants demonstrate approximate numerosity abilities from the first days of life^12^ and show early spatial-numerical mappings, associating smaller numerosities with left space and larger with right even when continuous magnitudes are controlled.^15^^,^^16^^,^^23^ Critically, infancy work demonstrates number-action mapping at the level of manual parameters: congruent numerosity-hand-aperture pairings elicit distinct electrophysiological responses compared to incongruent mappings,^23^ and infants detect violations of “natural” mappings more reliably than the reverse, suggesting early constraints that favor aligned sensorimotor correspondences.^21^ Together, these studies indicate that numerical magnitude is available early in a format compatible with spatial and attentional coding that may provide a foundation upon which number–action interactions can later emerge through sensorimotor development.

Childhood then reveals how action-based routines shape the internal structure of number. Finger gnosis and FMSs predict counting and arithmetic performance,^32^^,^^41^ and visuomotor integration is strongly associated with preschool mathematics.^40^ Critically, children transition from counting physical objects to finger-based routines to internalized representations, and finger-based “hand structures” can persist as cognitive signatures: children’s split-five effects reveal an implicit five-based hand-chunk structure that reflects finger counting habits,^36^ with related effects persisting into adulthood and re-emerging under conditions that constrain finger use.^38^ These findings support “redeployment” and embodied-development accounts: sensorimotor routines do not merely support early performance; they may become compressed into internal representational priors that remain available long after overt action is no longer required.^32^

Importantly, childhood evidence also shows limits and specificity. Finger gnosis effects, while reliable, may explain a modest portion of variance in later arithmetic once general abilities are controlled,^44^^,^^45^ and task-specific patterns show that finger-related measures predict finger-based counting/calculation more than number-line estimation.^41^ This nuance is theoretically informative: it suggests that number-action coupling is not a global “motor advantage,” but a set of selective linkages that support different (or a variety of) representational formats and strategies.

(4) Adulthoods and aging suggest persistence with reweighting: integration, specificity, compensation.

During childhood, the sensorimotor system is functional to the development of numerical and mathematical abilities, determining the emergence of motor-based internal numerical representations which persist in adulthood. However, how does this link is shown in adulthood? What are the factors determining variability in this number-action associations? One hypothesis, currently largely unexplored, suggests that in the adult number processing shows progressive independence (e.g.,^122^). Recent neuroimaging data are in line with this view, showing that in the left superior parietal area, grasping-related neural activity correlated with the participants’ performance in calculation tasks, indicating increasing functional specificity in those participants with better performance.^93^ This result deserves further investigation and highlights the importance of considering individual variability in the study of number-action associations, and of embodied cognition in general.^123^

The aging literature reviewed here, though limited, suggests that number-action integration persists and is reweighted by changes in executive control, motor constraints, and compensatory neural recruitment. Executive functions—planning, inhibition, strategic reasoning—decline with age and rely on frontal regions vulnerable to aging.^99^^,^^100^^,^^101^^,^^124^ Because numerical performance is tightly intertwined with working memory and inhibitory control,^104^^,^^125^ aging may change the control and deployment of numerical representations more than the representations themselves. This aligns with mixed evidence on ANS aging^14^^,^^104^^,^^105^ and with the common pattern of preserved (or even improved) accuracy but slowed response times in symbolic tasks.^105^^,^^106^^,^^107^^,^^108^

Brain imaging evidence shows that older adults often recruit more bilateral and widespread networks during numerical tasks, interpreted as a sign of compensatory activity.^118^^,^^119^ Motor behavior also changes in ways that could modulate the influence of number-action coupling: older adults show slower prehension, wider apertures, and altered planning strategies,^112^^,^^113^^,^^114^^,^^115^^,^^126^ alongside broader motor network recruitment that may support adequate performance.^127^ Importantly, evidence from dual-task paradigms suggests preserved integration between goal directed motor and numerical processes in older adulthood: although older adults recruit more extensive motor networks, combined visuomotor and arithmetic tasks do not yield disproportionate performance decrements,^80^ and engaging in whole-body movement such as walking may slow arithmetic processing without compromising accuracy.^42^

These results are consistent with the idea that shared fronto-parietal resources can support coordination rather than guaranteeing interference in aging.

Finally, intervention evidence—while still limited-points to applied implications of an embodied, shared-systems view: for instance, abacus training, which couples fine motor routines with numerical manipulation and imagery, improves executive functions and numerical working memory in older adults.^81^ This suggests that re-engaging sensorimotor numerical routines may strengthen both domain-specific (number) and domain-general (control) processes, consistent with the broader claim that number-action coupling reflects a control-oriented architecture that can be harnessed therapeutically.^128^

## Author contributions

Conceptualization, E.N. and M.R.; literature search, S.P., M.G., S.N., M.S.S.; writing – original draft, S.P. and E.N.; writing – review and editing, all authors; visualization, M.G. and E.N.; supervision, E.N. All authors have read and agreed to the published version of the manuscript.

## Declaration of interests

The authors declare no competing interests.
